# Learning Health Systems Symposium: Charting the Future of Saskatchewan Healthcare

**DOI:** 10.1002/lrh2.70060

**Published:** 2026-01-07

**Authors:** Charlene Haver, Amir Reza Azizian, Christina Weise, Miranda Cary, Maggie King, Susan Shaw, Kyla Avis, Emiliana Bomfim, Beliz Açan Osman, Gary Groot

**Affiliations:** ^1^ Saskatchewan Centre for Patient‐Oriented Research Saskatoon Saskatchewan Canada; ^2^ Department of Learning Health Sciences University of Michigan Ann Arbor Michigan USA; ^3^ Stewardship and Clinical Appropriateness, Regina General Hospital, Saskatchewan Health Authority Regina Saskatchewan Canada; ^4^ Patient Partner North Battleford Saskatchewan Canada; ^5^ Saskatchewan Health Authority Saskatoon Saskatchewan Canada; ^6^ KDA Consulting Saskatoon Saskatchewan Canada; ^7^ Saskatchewan Health Quality Council Saskatoon Saskatchewan Canada; ^8^ Community Health and Epidemiology Department, College of Medicine University of Saskatchewan Saskatoon Saskatchewan Canada

**Keywords:** healthcare, learning health systems, patient‐oriented research, quality improvement

## Abstract

**Introduction:**

This article shares insights from the Learning Health Systems Symposium, “Charting the Future of Saskatchewan Healthcare: Generating Value within our Health Systems.”

**Methods:**

Patient Partners and over 100 professionals in the fields of research, healthcare, and policy joined the Saskatchewan Centre for Patient‐Oriented Research (SCPOR) in Regina, Saskatchewan Canada, for a day filled with knowledge about learning health systems (LHSs). Attendees provided key insights on how to build provincial capacity in LHSs and thereby improve the health of people in Saskatchewan.

**Results:**

Key insights from the symposium included strengthening meaningful partnerships with patients and the community; establishing a shared vision for LHSs; harmonizing conflicting priorities; removing silos; recognizing the natural tensions between academia and the healthcare system; and building on and aligning infrastructure to support LHSs development.

**Conclusion:**

The LHSs symposium provided a way for health system leaders, Patient Partners, researchers, clinicians, and students to develop a common understanding of LHSs and envision a future for LHSs in Saskatchewan, Canada. Key next steps were to strengthen patient and community engagement, work collaboratively to establish a shared vision of LHSs, better understand existing assets to support LHSs, and identify opportunities to build future LHSs infrastructure.

## 1 Introduction

Learning health systems (LHSs) enhance both individual and population health by combining discovery and implementation through iterative cycles [1]. An LHS cycle begins with the formation of a multi‐interest holder (i.e., individuals with legitimate interests in the issue) learning community [1, 2]. Led by the community, the process involves gathering data to understand current practices (referred to as practice to data); analyzing these data to create evidence for potential improvements (known as data to knowledge); combining locally sourced evidence with pertinent evidence from other sources and implementing interventions based on this evidence to achieve better outcomes (called knowledge to practice) [1, 3]. Consequently, the operational decisions and strategic planning in care delivery are informed by research and evidence perspectives, internal and external to organizations or health systems [4]. Throughout all the stages of the cycles, equity is a central priority and ‘gear’ that powers successful LHSs [2, 4].

LHSs gained interest in Canada when the Canadian Institutes of Health Research (CIHR) Strategy for Patient‐Oriented Research (SPOR) introduced LHSs as a core component area for the second grant call, a 5‐year extension of support for the SPOR Support for People and Patient‐Oriented Research and Trials (SUPPORT) Units, specialized research service centers spread geographically across Canada [5]. The overall aim of the SPOR initiative is to improve health outcomes and enhance patients' health through integration of evidence at all levels in the healthcare system [5]. CIHR defines patient‐oriented research as engaging the patient, caregivers, and families as partners in the research process, which helps to ensure research focuses on patient‐identified priorities, leading to improved patient outcomes [5].

The Saskatchewan health care system is made up of many provincial, regional, and local organizations working together to ensure healthy communities. The Minister of Health oversees the strategic direction of the healthcare system, and the Ministry of Health oversees and co‐ordinates the delivery of health services in the province [6]. The Saskatchewan Health Authority (SHA) is the provincial organization responsible for delivering the majority of publicly funded health services throughout the province and serves a diverse population of 1.2 million residents with over 40 000 employees and physicians [7]. Additionally, the SHA is a key partner of the Saskatchewan Centre for Patient‐Oriented Research (SCPOR). SCPOR is the provincial SUPPORT Unit, and as a result of the renewed LHS focus for SUPPORT Units, SCPOR set out to work directly with the SHA [8] to determine how to build and support LHSs. SCPOR and senior leaders in the SHA began working together to support building LHSs in the Saskatchewan health system in 2022. Rather than focus on one specialized population or problem, the main issue the Saskatchewan health system would like to use the LHS approach to address is fixing inefficiencies in the health system overall and improving patient experiences and outcomes. Over time, it became evident there was not a common understanding of LHSs within the SHA and across health system organizations in the province. To foster a common understanding and advance the development of LHSs in Saskatchewan, SCPOR and its provincial partners decided that SCPOR would host an LHS symposium.

On May 27, 2024, SCPOR hosted “Charting the Future of Saskatchewan Healthcare: Generating Value within our Health Systems,” which brought together members of Saskatchewan's health system to explore strategies for building and strengthening LHSs in the province. In this report, we provide a summary of the discussions and key insights

## 2 Methods

The symposium consisted of keynote presentations on LHSs from leaders across Canada and within Saskatchewan, followed by facilitated in‐person discussions. The LHS Action Framework [4], developed in Ontario, Canada, was presented, introducing the concept of supporting learning and improvement in health systems and demonstrating the three‐legged stool of system operators, Patient Partners, and evidence‐support providers. Later, the learning health and social system model implemented in Newfoundland and Labrador was shared, followed by a presentation of the Strategic Clinical Networks as an example of LHSs in Alberta [9, 10].

Following the presentations, attendees were divided into two break‐out groups to discuss the possibilities for the future of LHSs in Saskatchewan. Group A attendees were selected based on their roles as senior leaders in the provincial health system or as individuals with experience working as a Patient Partner with the Saskatchewan SUPPORT Unit, SCPOR, to allow for shared learning and alignment of vision to move LHSs forward. Group A included 30 individuals from the SHA, Saskatchewan Ministry of Health, Indigenous health system leaders, Saskatchewan Health Quality Council, Saskatchewan Cancer Agency, eHealth Saskatchewan, and Patient Partners. Group B consisted of all remaining attendees and included 70 individuals from organizations including the SHA, Saskatchewan Ministry of Health, Saskatchewan Health Quality Council, Saskatchewan Cancer Agency, eHealth Saskatchewan, SCPOR, as well as Patient Partners, researchers, and students. Senior leaders and staff from all health system organizations in the province attended the symposium, demonstrating high engagement at the provincial level of interest in advancing LHSs.

Trained facilitators led discussions with both groups.

- Groups A and B were asked: “What are the possibilities in Saskatchewan to foster learning health systems?” Responses were categorized as: Excitement, Opportunities, Strengths or Immediate Improvements. Attendees worked in sub‐groups of 6–10 people and recorded their responses, which were verbally shared with the other attendees and meeting facilitators.
- Members in Group A were also asked “What are the next steps for how to move forward with LHSs in Saskatchewan?”. Attendees worked in sub‐groups and reported on their responses within Group A.
- Group B was asked “If you were 10 times bolder, what big idea would you recommend to move LHSs forward in Saskatchewan?” Participants anonymously recorded individual responses, which were shared with other members within Group B. Participants rated each other's big ideas using a scale of 1–5 (1 for a low score and 5 for a high score), for a maximum total of 25 points after five rounds. Big ideas with the top scores were identified by the facilitators and then shared with all Group B attendees.

Principles of rapid qualitative research served as key methodological references for this report [11, 12, 13, 14, 15, 16]. Rapid and real‐time analysis is an efficient method to produce information quickly and effectively to support collaborative decisions and prompt delivery of findings. Unlike traditional qualitative research methods, which may require extensive time and resources, rapid qualitative analysis aims to provide timely insights that can inform decision‐making and drive action. Principles of the Rapid Insight (RI) method were also used to develop this report. RI is an approach developed by National Health Service (NHS) Horizons which turns data from highly interactive events involving large numbers of people, into knowledge that can be acted upon to positively affect change. RI is designed to extract actionable intelligence from large or complex datasets, such as those generated during events or workshops [17, 18]. RI is facilitated by qualitative and mixed methods research tools, which promote shared learning and collective understanding of the discussions at hand.

The RI process was implemented as follows: Themes from Groups A and B were captured from report‐out discussions, from a review of facilitators notes and rapid data collection following the RI methodology. Immediately following each discussion, field notes, including researchers', facilitators', and participants' written inputs, were consolidated into a structured data matrix. Researchers conducted a rapid content analysis by clustering similar responses and summarizing key patterns emerging across sub‐groups. An exhaustive cross‐review of field notes was conducted to confirm the consistency and completeness of key points before synthesis. Through this iterative, team‐based approach, individual reflections were aggregated into thematic summaries, from which the nine overarching insights were derived. Additionally, key statements were inductively coded in real time and categorized under pre‐defined domains (Excitement, Opportunities, Strengths, Immediate Improvements, and Bold Ideas) for Groups A and B.

## 3 Results

Key insights from the LHSs symposium were summarized and are provided in Table 1. Of note, not all recommendations that were given were clear and/or practical, came with a path for next steps, and/or how to achieve the recommendations. Nine key insights were categorized, with recommendations under each theme. A main theme identified was the need to strengthen meaningful partnerships with Patient Partners and the community. Attendees recommended listening to all voices through a trauma‐informed and equity lens, grounded in lived experiences. A second key insight was the necessity to establish a shared vision for LHSs in the province. Attendees vocalized a desire to work collaboratively to develop a shared vision with Patient Partners, the health system, and researchers to improve care. The third key insight focused on building upon, aligning, and strengthening existing LHS assets, such as positions (e.g., data analysts), software (e.g., REDCap), and/or methods (e.g., quantitative and qualitative data collection) that organizations currently utilize that support LHSs. Attendees noted there are many existing LHSs assets and resources that should be identified, strengthened, and integrated to build LHSs in Saskatchewan. The need to strengthen provincial governance and leadership was also identified, with recommendations to harmonize and recognize natural tensions. Finally, attendees recommended prioritizing patient‐centered metrics and listening to the experiences of patients by including patient‐reported experience measures (PREMs) and patient‐reported outcome measures (PROMs) at every level of the health system. For a complete list of insights and recommendations, see Table 1.

**TABLE 1:** LHSs symposium key insights.

Key insights to successfully advance LHS in Saskatchewan	Recommendations
Strengthen Meaningful Partnerships with Patients and Community	Listen to all voices and experiences through trauma informed and equity lenses.
	Embrace productive friction; ask mindful questions while avoiding burdensome surveys and consultations.
	Always ground efforts in lived experiences to produce better outcomes and experiences for all.
Establish a Shared Vision for Made‐in‐Saskatchewan LHSs	Work with Patient Partners, the wider health system, and researchers.
	Respond to the challenge of building LHSs to improve health and healthcare.
	Start this as a collaborative journey with an agreed upon destination.
Build on and Align our Strengths and Assets	Start from Saskatchewan's many existing assets: the pieces of the puzzle.
	Identify the assets, align them, connect them and integrate them.
	Embed the patient perspective throughout the process of “leveling up” our current learning for improvement strategies and efforts.
	Think and act as one, which is essential for this to work.
Strengthen Governance and Leadership	Harmonize conflicting priorities.
	Recognize the natural tensions between the academic ecosystem and the healthcare ecosystem.
	Set aside the wants and needs of each system and focus on what matters to patients and communities.
	Build a decision‐making/governance model where bold and innovative approaches can be effectively and efficiently advanced.
Leverage the Resources of SCPOR	Capitalize on SCPOR‘s position as a partnership of organizations where resources are held to support the integration of evidence to support patient‐oriented research.
	Ensure partners are identifying the priority investments to build infrastructure and capacity in the province.
Think Bigger. Think Broader	Use the LHSs approach to address urgent challenges, but don't lose sight of the bigger picture or the rapidly approaching future.
	Consider the multi‐sectoral, interconnected systems that produce health and wellness.
	Act on immediate needs while also designing long‐term, sustainable changes to those systems failing to produce desired outcomes within existing foundations and structures.
Prioritize Patient‐Reported Metrics	Listen to the experiences of patients by including patient‐reported experience measures (PREMs) and patient‐reported outcome measures (PROMs) at every level of the health system.
	Overcome resistance to data for learning by elevating the voice of the people of Saskatchewan in our conversations.
Diversify How We Utilize Data	Recognize that data are not just numbers within reports or dashboards; data for learning are generated in every interaction with every patient and every community.
	Continue to build our quantitative data systems but also consider the valuable information we can ascertain using other methodologies to drive learning.
Collaborate Courageously	Have difficult conversations to generate the energy for change; one person's comfort is another's discomfort.
	Commit to work through the conflicts and tensions that inevitably arise in complex system change.
	Learn to be comfortable with the uncomfortable in all shared spaces.

Group A and B discussions about the possibilities to foster LHSs in Saskatchewan resulted in 61 unique and 19 overlapping reflections (Table 2). When asked what excites participants about LHSs, Group A highlighted: alignment and collaboration, opportunities for specific focus areas, system integration, and transparency. Group B highlighted: patient‐centered focus, awareness and connection, building capacity, and integration of PROMs and PREMs in the health system. Overlapping reflections between the two groups included engagement and inclusivity, virtual care initiatives, knowledge mobilization, and learning from others.

**TABLE 2:** Possibilities in Saskatchewan to foster LHSs.

Category	Group A reflections	Group B reflections	Overlapping reflections between Groups A and B
Excitement	Enhanced approaches and innovations	Patient‐centered care focus	Engagement and inclusivity
	Alignment and collaboration	Awareness and connection	Virtual care initiatives
	Learning health system potential	Individual research projects	Knowledge mobilization
	Opportunity for specific focus areas	Health system support for research	Learning from others
	System integration and transparency	Rural/remote community engagement	
	Virtual health and point of care	Equity and commitment	
	Broader perspectives and metrics	Capacity building	
	Commitment to full implementation	Integration of PREMs and PROMs	
		Community‐led innovations	
Opportunities	Governance and workforce engagement	Scientific expertise embedded in primary care networks	Investment in data infrastructure
	Infrastructure and process development	Broad system focus	Engagement with indigenous peoples
	Enhanced decision making	Partnerships with data holders	Measurement of impact
	Role clarification and prioritization	Collection of patient‐centered metrics	Data integration and accessibility
	Learning Health System Framework	Sustainable patient‐oriented research	Culturally responsive care
	Embedded research and skills	The scaling up of successful innovations	
		Value‐based care improvement	
		Collaboration with other provinces	
		Improved information flow and inter‐organizational collaboration	
Strengths	Unified systems	Patient‐centered measurement	Patient partner engagement
	Integration and communication	Electronic health records	Cooperation among multiple partners
	Learning and adaptation	Health system support for research	Community commitment and collaboration
	Opportunities for growth	Data availability and expertise	Existing infrastructure, expertise and human resources
		Engagement of talent and interest‐holders	
		Support for research	
		Quality improvement maturity	
		Diverse skillsets	
		Community‐driven collaboration	
Immediate improvements	Infrastructure investment	Patient‐centered framework	Clear and shared vision for LHSs
	Data and information systems	Decision‐maker debriefs	Improved governance structure and engagement
	Strategic planning and alignment	Shared event information	Enhanced communication and transparency
	Focus on outcomes and metrics	Survey evaluation	Leadership and governance
		Patient‐identified research	Collaborative efforts and partnerships
		Research funding pipeline	Removal of silos
		Current state assessment	
		Accountability and transparency	
		Space for collaboration and innovation	
		Resource management and realignment	
		Communication and engagement	

When asked about the opportunities to foster LHSs (Table 2), Group A indicated opportunities exist in governance and workforce engagement, infrastructure and process development, and enhanced decision‐making. Group B shared patient‐centered metrics, value‐based care, and sustainable patient‐oriented research as opportunities to foster LHSs. Overlapping opportunities identified by both Groups A and B were investment in data infrastructure, engagement with Indigenous Peoples, measurement of impact, data integration and accessibility, and culturally responsive care.

For LHSs strengths (Table 2), Group A indicated unified systems, integration, and communication, as well as learning and adaptation to foster LHSs, whereas Group B identified patient‐centered measurement, electronic health records, and research supports as strengths. Overlap between both groups included Patient Partner engagement, cooperation among partners, community collaboration, and utilization of existing infrastructure.

Within the category of immediate improvements to foster LHSs (Table 2), Group A highlighted the need for infrastructure investment, data and information systems, strategic planning and alignment, and a focus on outcomes and metrics. Group B identified immediate improvements made by a current state assessment, communication and engagement, and space for collaboration and innovation. Overlap between Groups A and B included the establishment of a shared LHSs vision, improved governance structure and engagement, enhanced communication and transparency, collaborative partnerships, as well as the removal of silos.

Unique to Group B was the “bold ideas” activity where attendees were asked “If you were 10 times bolder, what big idea would you recommend?” The top eight ranked ideas from attendees are shared. The top three ideas, with 25 points each, included dedicated resources and engagement of senior leadership in the health system, commitment of health system leaders with content experts to discuss how to incorporate learnings into practice, and break down of silos. The next top ranked ideas, with a total of 23 points each, were to consider equity and the value of all system members, to make the most out of what we have, and to incorporate ideas without judgment. Lastly, the two ideas with 22 points each were to meaningfully engage interest‐holders to identify priorities and to collaborate with provincial partners.

Group A was uniquely tasked with identifying next steps to move LHSs forward in the province. Group A recommended creating a provincial LHS asset map, using the LHS Gears Framework [4] as a guide to better understand and integrate provincial LHS activities. Following the asset mapping exercise, Group A indicated the province should use the learnings from the asset map and convene provincial health system leaders to discuss opportunities to move LHSs forward. Lastly, it was recommended to continue focusing on the standardized provincial implementation of PROMs and PREMs, identifying what is working well, spreading and scaling things that are working, and addressing what is not working well.

## 4 Discussion

The LHSs Symposium provided a way for health system leaders, Patient Partners, researchers, clinicians, and students to develop a common understanding of LHSs and envision a future for LHSs in Saskatchewan. Overall, the findings that came from the symposium are fundamentals of a high quality, highly effective and efficient health system and reflect the state of the health system context in Saskatchewan. Based on the findings from the LHSs Symposium, it was evident that patient and community engagement is essential to building LHSs. One of the key insights from the symposium was the need to strengthen meaningful partnerships with Patient Partners and the community. In the LHS Action Framework Model by Reid et al. [4], the patient, caregiver, and provider are located in the middle of the model, demonstrating they are the central components of LHSs. Direct engagement and co‐design with the people impacted by the healthcare issues, along with those who influence them, are needed for successful implementation [4]. By incorporating those with lived experiences, grounding efforts in real‐world insights, and ensuring all voices are heard and valued, the goal of enhanced health outcomes is more achievable.

An overarching theme that emerged from the symposium was the need for inclusivity, community commitment, and collaboration when building LHSs. Opportunities to engage diverse groups, including Indigenous Peoples, and opportunities to deliver culturally responsive care were noted as key to building LHSs. This idea connects to the internal driver of equity in the LHS Action Framework, where Reid et al. [4] state equity is attained when health system disparities are eliminated across groups with differing levels of social advantage and disadvantage. LHS activities must be inclusive of, and create value for, equity deserving groups. A key next step to building LHSs is to be inclusive of the diverse voices of the Patient Partners and community members to continue to strengthen engagement. This may be achieved by meaningfully including Patient Partners and community members at the onset of and throughout research projects, incorporating their identified priorities, and establishing and nurturing relationships. To ensure equity, diversity, and inclusion are incorporated into the next steps of the LHS work in Saskatchewan, SCPOR's Indigenous Health Advisory Council, Patient Partners, and Indigenous health system leaders, as well as members of other equity‐deserving populations in the health system, will be included in strategic conversations and shared visioning for building LHSs in the province.

Establishing a shared vision to build LHSs was a key take‐away from the symposium. Working with Patient Partners, communities, the health system, researchers, and clinicians to create a collective vision to improve the health of others is essential. Meaningful interest‐holder engagement was highlighted as essential for identifying priorities, implementing strategies, and establishing performance indicators, emphasizing the need for all partners to align on common issues. Capitalizing on SCPOR's position as a SPOR SUPPORT Unit and its provincial partnerships can help align the LHSs vision and support patient‐oriented research to produce better patient outcomes.

Attendees at the symposium were excited about the opportunity to collaborate with provincial partners and respond to the challenge of building LHSs to improve the health system. However, misalignment of vision is a common challenge when developing LHSs. Ongoing tensions in LHSs among interest‐holders, including researchers, health system leaders, and funders, are common and were raised by Reid and Greene [19]. Health research and health systems are often disconnected, which can impede the rapid learning process [19]. The LHSs Symposium highlighted the need to harmonize conflicting priorities, remove silos, and recognize the natural tensions between academia and the healthcare system. Attendees identified there is a need to have difficult conversations to generate energy for change and a need to commit to working through the tensions that arise in complex system change. The focus should be on what matters most to Patient Partners and families. Identified opportunities for immediate improvements included removal of silos, enhanced communication and transparency, commitment of health system leaders with content experts, as well as collaborative efforts and partnerships.

Infrastructure development is a crucial component that is needed to advance LHSs. Attendees identified existing infrastructure to support LHSs, including electronic health records, data availability, research expertise, and existing human resources as strengths in the province. Opportunities to build additional LHSs infrastructure included virtual care initiatives, further investment in data infrastructure, and integration of PROMs and PREMs. One way to further invest in data infrastructure is by continuing to support SCPOR's Health Research Data Platform—Saskatchewan (HRDP‐SK), which is the province's first fully integrated multi‐agency data access platform for health research [20]. The HRDP‐SK is a critical resource for LHSs in Saskatchewan because it leads to more efficient and effective research by giving researchers timely, coordinated access to health data. This key LHS resource will be connected to the PREMs and PROMs work in the future.

There is a need to listen to the experiences of patients by including PROMs and PREMs at every level of the health system, as PROMs and PREMs are becoming essential parts of LHSs [21]. Data are generated in every patient interaction and can be used to create LHSs where we learn from every patient who is treated [22]. Future recommendations included standardizing provincial implementation of PROMs and PREMs and gaining an understanding of existing provincial LHSs infrastructure by building a provincial LHS asset map of existing assets (e.g., positions, skillsets, and technological systems), which aligns with the recommendations in the literature to assess and align the resources that exist to enable LHSs [19, 23]. SCPOR and the attendees of the LHS Symposium will subsequently aim to collaboratively use the asset map to identify resources that could support LHSs, better understand strengths, and identify gaps and opportunities for future investment to build future LHSs infrastructure.

## 5 Conclusion

The LHSs Symposium provided a way for health system leaders, Patient Partners, researchers, clinicians, and students to develop a common understanding of LHSs and envision a future for LHSs in Saskatchewan. Engaging Patient Partners and community was identified as essential to building LHSs, as was establishing a shared vision among Patient Partners, the health system, and researchers within the province. SCPOR will continue to work with its partners, attendees of the symposium, and people with lived experience to leverage existing strengths and partnerships, better understand existing infrastructure and how to elevate assets, and invest in future infrastructure. As a result, LHSs would be further strengthened and lead to improved outcomes for patients in Saskatchewan.

## Funding

The Saskatchewan Centre for Patient‐Oriented Research (SCPOR) is funded by the Canadian Institutes of Health Research (CIHR).

## Conflicts of Interest

The authors declare no conflicts of interest.

## Data Availability

The data that support the findings of this study are available from the corresponding author upon reasonable request.

## References

[1] C. P. Friedman , “What Is Unique About Learning Health Systems?,” Learning Health Systems 6, no. 3 (2022): e10328. 35860320 10.1002/lrh2.10328 PMC9284922

[2] C. P. Friedman , E. A. Lomotan , J. E. Richardson , and J. L. Ridgeway , “Socio‐Technical Infrastructure for a Learning Health System,” Learning Health Systems 8, no. 1 (2024): e10405. 38249851 10.1002/lrh2.10405 PMC10797563

[3] M. Menear , M. A. Blanchette , O. Demers‐Payette , and D. Roy , “A Framework for Value‐Creating Learning Health Systems,” Health Research Policy and Systems 17, no. 1 (2019): 79. 31399114 10.1186/s12961-019-0477-3 PMC6688264

[4] R. J. Reid , W. P. Wodchis , K. Kuluski , et al., “Actioning the Learning Health System: An Applied Framework for Integrating Research Into Health Systems,” SSM ‐ Health Systems 2 (2024): 100010.

[5] Canadian Institutes of Health Research Government of Canada , Canada's Strategy for Patient‐Oriented Research ‐ CIHR [Internet], 2011, https://cihr‐irsc.gc.ca/e/44000.html#a4.4.5.

[6] Government of Saskatchewan , Understanding the Health Care System, Health Care Information in Saskatchewan, https://www.saskatchewan.ca/residents/health/understanding‐the‐health‐care‐system.

[7] Our Organization | SaskHealthAuthority, [Internet], https://www.saskhealthauthority.ca/our‐organization.

[8] Saskatchewan Centre for Patient‐Oriented Research [Internet] , Our Story, https://www.scpor.ca/our‐story.

[9] NL SUPPORT [Internet] , Learning Health and Social System, https://nlsupport.ca/about/learning‐health‐and‐social‐system/.

[10] Alberta Health Services , Strategic Clinical Networks, https://www.albertahealthservices.ca/scns/scn.aspx.

[11] J. Beebe , Rapid Assessment Process: An Introduction (Bloomsbury Academic, 2001), https://rowman.com/ISBN/9780759100114/Rapid‐Assessment‐Process‐An‐Introduction.

[12] G. A. Johnson and C. Vindrola‐Padros , “Rapid Qualitative Research Methods During Complex Health Emergencies: A Systematic Review of the Literature,” Social Science & Medicine 189 (2017): 63–75. 28787628 10.1016/j.socscimed.2017.07.029

[13] A. L. Nevedal , C. M. Reardon , M. A. Opra Widerquist , et al., “Rapid Versus Traditional Qualitative Analysis Using the Consolidated Framework for Implementation Research (CFIR),” Implementation Science 16, no. 1 (2021): 67. 34215286 10.1186/s13012-021-01111-5 PMC8252308

[14] C. Vindrola‐Padros , Doing Rapid Qualitative Research [Internet] (Sage Publications, 2021), https://www.barnesandnoble.com/w/doing‐rapid‐qualitative‐research‐cecilia‐vindrola‐padros/1138982387.

[15] Sage Research Methods Community [Internet] , What is Rapid Qualitative Research?, 2021, https://researchmethodscommunity.sagepub.com/blog/what‐is‐rapid‐qualitative‐research.

[16] C. Vindrola‐Padros and G. A. Johnson , “Rapid Techniques in Qualitative Research: A Critical Review of the Literature,” Qualitative Health Research 30, no. 10 (2020): 1596–1604. 32667277 10.1177/1049732320921835

[17] S. Karakusevic , L. Yearsley , and L. Maddocks‐Brown , “Networks, the Clear Blue Water of Change or the “Wave Tops” on the Sea?,” Healthcare Management Forum 37, no. 3 (2024): 164–167. 37971279 10.1177/08404704231211163

[18] NHS Horizons [Internet] , Rapid Insight: The Missing Piece in How We Lead Large‐Scale Change, 2024, https://horizonsnhs.com/rapid‐insight‐the‐missing‐piece‐in‐how‐we‐lead‐large‐scale‐change/.

[19] R. J. Reid and S. M. Greene , “Gathering Speed and Countering Tensions in the Rapid Learning Health System,” Learning Health Systems 7, no. 3 (2023): e10358. 37448454 10.1002/lrh2.10358 PMC10336490

[20] Saskatchewan Centre for Patient‐Oriented Research [Internet] , Health Research Data Platform ‐ Saskatchewan (HRDP‐SK), https://www.scpor.ca/hrdpsk.

[21] V. Lowry , V. Tremblay‐Vaillancourt , P. Beaupré , et al., “How Patient‐Reported Outcomes and Experience Measures (PROMs and PREMs) are Implemented in Healthcare Professional and Patient Organizations? An Environmental Scan,” Journal of Patient‐Reported Outcomes 8, no. 1 (2024): 133. 39546094 10.1186/s41687-024-00795-9 PMC11568099

[22] T. Foley and L. Vale , “A Framework for Understanding, Designing, Developing and Evaluating Learning Health Systems,” Learning Health Systems 7, no. 1 (2023): e10315. 36654802 10.1002/lrh2.10315 PMC9835047

[23] V. C. Rentes , C. Kalpakjian , A. Sales , and A. Krumm , “Operationalizing a Learning Health System: A Self‐Assessment Tool for Interprofessional Teams,” Learning Health Systems 9, no. 3 (2025): e10482. 40677603 10.1002/lrh2.10482 PMC12264368

