# Correction to “Learning Health Systems Symposium: Charting the Future of Saskatchewan Healthcare”

**DOI:** 10.1002/lrh2.70081

**Published:** 2026-04-26

C. Haver , A. R. Azizian , C. Weise , et al., “Learning Health Systems Symposium: Charting the Future of Saskatchewan Healthcare,” Learning Health Systems 10, no. 1 (2026): e70060, 10.1002/lrh2.70060.41573594 PMC12819043

In the above‐mentioned article, the authors would like to add further information regarding the funding. The updated Funding statement is as follows:

“The Saskatchewan Centre for Patient‐Oriented Research (SCPOR) is funded by the Canadian Institutes of Health Research (CIHR), funding number SS2‐177512.”

We apologize for this error.

